# Relationship between anaerobic power and start performance parameters in adolescent swimmers

**DOI:** 10.1186/s13102-026-01583-2

**Published:** 2026-02-11

**Authors:** Yücel İnaç, Şaban Ünver, Emre Şimşek, Hayati Arslan, Doğan Altun, Anıl Ulaç Atan, Tülin Akman

**Affiliations:** 1 Faculty of Yasar Doğu Sport Sciences, Ondokuz Mayıs University, Samsun, 55270 Türkiye; 2 Faculty of Sports Science, Erciyes University, Kayseri, 38280 Türkiye

**Keywords:** Anaerobic power, Swimming, Adolescents, Vertical jump, Start performance

## Abstract

**Background:**

Start performance is a critical determinant of success in competitive swimming. This study investigated the relationships between anaerobic power (AP), vertical jump height (VJ), and flight distance (FD) in adolescent swimmers.

**Methods:**

Fifty-four Tier 2 adolescent swimmers (32 males, 22 females; age: 13.57 ± 1.31 years) participated in the study. Anaerobic power was calculated using the Lewis formula, and FD was measured via video analysis with Kinovea software. Statistical analyses included independent samples t-tests, Pearson correlations, and multiple regression models to identify performance predictors.

**Results:**

Significant gender differences were observed in body height, weight, VJ, FD, and AP (all *p* < 0.05), with males outperforming females, while training experience was similar. FD showed a significant positive correlation with AP (*r* = 0.51, *p* < 0.001), representing a moderate effect size. Multiple regression analysis further revealed that Age (β = 0.65, *p* < 0.001) and Gender (β = 0.32, *p* = 0.003) were the primary predictors of FD, while AP did not emerge as an independent predictor in the final model.

**Conclusion:**

Although anaerobic power is associated with start performance, age and gender are the strongest predictors of flight distance in adolescent swimmers. These findings add to previous research by highlighting the importance of maturational and technical development for optimizing start efficiency during early specialization.

## Introduction

Swimming is one of the most widely practiced aquatic sports worldwide, known for developing endurance, flexibility, and muscular strength while minimizing joint stress due to the buoyancy of water [1]. Beyond its health benefits, swimming performance relies heavily on technical, physiological, and biomechanical factors, particularly in short-distance events (< 100 m) where explosive power and start performance play a decisive role [2]. From a biomechanical perspective, swimming is a complex discipline, as performance is influenced not only by the athlete’s movements but also by hydrodynamic and aerodynamic principles [3].

The sprint start is a critical determinant of success in competitive swimming, as even small differences in start performance can significantly influence race outcomes [2]. Research shows that start performance (e.g., time to 15 m) can comprise up to 30% of total race time in 50 m freestyle events [4]. Among the determinants of start effectiveness, vertical jump (VJ) performance is widely used as an indirect indicator of lower-limb explosive strength and anaerobic power (AP) [5, 6]. These capacities are crucial for achieving high take-off velocity and longer flight distance (FD) during swim starts [7].

Previous biomechanical research suggests that the transfer of land-based power to aquatic performance arises from a functional continuity between lower-limb force generation and start dynamics [4, 5, 8, 9]. While several studies have demonstrated these relationships in elite adult swimmers, recent research has further explored the role of leg drive and block kinematics in high-performance athletes [10–12]. However, limited evidence exists regarding these relationships in adolescent populations. During the start phase, swimmers execute a rapid and forceful push-off from the block that biomechanically parallels a vertical jump in terms of joint sequencing, rate of force development, and neuromuscular coordination. Accordingly, indices of explosive leg power such as VJ height and calculated AP are expected to predict take-off velocity and FD, thereby contributing to overall start efficiency.

Although previous studies have demonstrated relationships between AP, VJ, and FD in elite swimmers [4, 9], these investigations have primarily focused on adult or high-performance athletes. The developmental stage of adolescence introduces unique variables, such as rapid changes in anthropometrics and maturation, which may alter the established power-performance relationships seen in adults. Currently, there is a lack of research explicitly investigating how land-based power indicators jointly relate to FD specifically in adolescent swimmers during early specialization. In the present study, flight distance was selected as the primary outcome variable because it represents the immediate mechanical consequence of force application during the start phase, largely independent of underwater kicking proficiency and stroke-specific technical skills. In adolescent swimmers, temporal start variables such as time to 5–15 m may be confounded by differences in underwater technique, coordination, and training experience. Therefore, flight distance was prioritized as a more direct and biomechanically grounded indicator of start-phase propulsion. Therefore, the present study aimed to examine these relationships in a youth population, providing new insight into early performance development and implications for talent identification and training design.

Based on the theoretical and biomechanical framework described above, it was hypothesized that:

- H_1_: Higher AP is associated with longer FD during the start phase.
- H_2_: Better VJ performance is associated with longer FD during the start phase.

Additionally, anthropometric factors such as height and body mass were expected to contribute significantly to start performance variability due to their known influence on take-off velocity and propulsion.

## Material and method

### Participants

Fifty-four competitive swimmers (32 males, 22 females; mean age = 13.57 ± 1.31 years) participated in the study. Following the Participant Classification Framework [13], swimmers were categorized as Tier 2 (Trained/Developmental). This classification is significant for the study as it ensures that participants are at a comparable competitive level and follow standardized training volumes, minimizing performance variance due to vastly different training backgrounds.

Inclusion criteria required at least five years of competitive experience and no musculoskeletal injuries in the previous 12 months. Biological maturation was not clinically assessed (e.g., Tanner stages); however, all participants were in the adolescent growth period, and chronological age was used as a proxy for developmental stage. Anthropometric measurements were obtained using a calibrated Seca 877 electronic scale. The study was approved by the Ondokuz Mayıs University Clinical Research Ethics Committee (No: 2023/672) and conducted per the Declaration of Helsinki.

### Study design

This cross-sectional study was conducted in a 50 m indoor short-course pool with a standardized depth of 2 m. All tests were performed on the same day to minimize variability. Participants first completed the vertical jump test, followed by the flight distance test after a standardized warm-up.

### Procedure of the study

Testing occurred at the Samsun Atakum Olympic Swimming Center (water temp: 26–28 °C; ambient temp: 31–32 °C). To control for circadian variation, sessions were conducted between 16:00–18:00. The 10-minute dry-land warm-up included specific dynamic stretches (leg swings, arm circles, lunges) and two sets of three submaximal countermovement jumps.

All swimmers used a standardized track start procedure on Omega OSB11 starting blocks, which were configured identically for all participants to ensure fair measurement of takeoff mechanics.

### Measurement of vertical jump (VJ)

Vertical jump height was assessed using a Takei 5406 digital jump meter (Takei Scientific Instruments, Japan), which calculates jump height based on flight time. Participants performed the countermovement jump (CMJ) starting from an upright standing position. To isolate lower-limb explosive power and eliminate the influence of upper-body momentum, participants were instructed to keep their hands on their hips throughout the entire movement. Each participant performed three maximal trials with 5-minute rest intervals between attempts to ensure full recovery. The best (highest) value was recorded for statistical analysis. The test-retest reliability for this protocol was excellent (ICC = 0.95, 95% CI = 0.91–0.98).

### Measurement of flight distance (FD)

Flight distance was analyzed using Kinovea software (version 0.9.5). A high-definition camera (iPad, Full HD, 60 fps) was positioned 3 m from the starting block, strictly perpendicular to the swimmer’s trajectory to minimize parallax error. Digital calibration was performed using a two-point method with a 1-meter reference scale object placed in the same sagittal plane as the swimmer’s flight path. To ensure precise identification of the water entry point, all swimmers wore standardized swimming caps and goggles. Flight distance (FD) was defined as the horizontal distance from the edge of the starting block to the point where the fingertips first contacted the water. The inter-rater reliability for the FD measurement was excellent (ICC = 0.94, 95% CI = 0.89–0.97).

### Anaerobic power calculation (AP)

Anaerobic power (W) was estimated using the Lewis formula, which integrates body mass and vertical jump height [14]. Although originally developed by Fox and Mathews [15], the formula has been validated in youth populations and used in similar studies examining explosive power in swimmers [7, 9].

$${P}\left({Watts}\right)=\;\sqrt{4.9}\;\times\;{body\;mass (kg)}\;\times\;\sqrt{{jump\;height (m)}}\;\times\;9.81$$

### Statistical analysis

All analyses were performed using SPSS (version 21.0). Normality of data was assessed with the Shapiro-Wilk and Levene’s tests. Between-group differences were examined using independent-samples t-tests with effect sizes (Cohen’s d) and 95% confidence intervals. Pearson correlation coefficients with 95% confidence intervals were used to assess relationships between FD, VJ, and AP. To address the rationale for including age, height, and weight in the regression model, potential multicollinearity was monitored using Variance Inflation Factor (VIF) values, ensuring all VIF < 2.0 to maintain model stability. Statistical significance was set at *p* < 0.05. An a priori power analysis was performed using G*Power version 3.1.9.7 to determine the minimum required sample size for correlation analyses. Assuming a medium effect size (*r* = 0.40), a two-tailed α level of 0.05, and a statistical power (1–β) of 0.80, the required sample size was calculated as *N* = 47. The final sample of 54 swimmers therefore exceeded this threshold, providing adequate statistical power for detecting medium-to-large effects.

## Results

The descriptive characteristics of the participants, including age, body height, body weight, and training experience, are summarized below Table 1. Overall, male swimmers were taller and heavier than females, whereas training experience did not differ significantly between groups (*p* > 0.05).

**Table 1:** Descriptive characteristics of swimmers

	Male (*n* = 32) Mean ± SD	Female (*n* = 22) Mean ± SD	Cohen’s d	*p*-value
Age (years)	13.71 ± 1.40	13.36 ± 1.18	0.27	0.270
Height (cm)	166.62 ± 10.13	157.59 ± 8.09	0.97	0.004
Body Weight (kg)	56.28 ± 13.12	47.18 ± 8.07	0.82	0.011
Sports Age (years)	6.09 ± 1.55	6.18 ± 1.86	-0.05	0.860

Performance characteristics by gender Table 2. Male swimmers showed higher AP, FD, and VJ values, all statistically significant with moderate to large effect sizes.

**Table 2:** Performance characteristics of swimmers

	Male Mean ± SD	Female Mean ± SD	Cohen’s d	*p*-value
AP (w)	728.96 ± 178.79	584.59 ± 114.62	0.95	0.002
FD (cm)	351.89 ± 58.96	303.47 ± 47.96	0.87	0.002
VJ (cm)	35.60 ± 4.39	32.54 ± 4.18	0.71	0.013

As shown in Table 3, a statistically significant relationship was detected between AP and FD, VJ, age, height and body weight (*p* < 0.001). FD was also positively correlated with age, height and body weight (*p* < 0.001), while VJ was correlated with height (*p* < 0.05).

**Table 3:** Correlations between performance and anthropometric variables

Variable Pair	*r*	*p*
AP ↔ Age	0.575	0.001
AP ↔ Height	0.868	0.001
AP ↔ Body Weight	0.960	0.001
FD ↔ Age	0.691	0.001
FD ↔ Height	0.652	0.001
FD ↔ Body Weight	0.593	0.001
VJ ↔ Height	0.293	0.043

To isolate the influence of explosive power from anthropometric growth, partial correlation analyses were conducted while controlling for body mass and height. The results revealed that AP showed a trend toward significance, while VJ remained significantly associated with FD. These findings suggest that VJ height provides a slightly more stable land-based indicator of FD than calculated AP when body size is accounted for (Table 4).

**Table 4:** Partial correlations between FD and performance variables controlling for body mass and height

	Partial *r*	*p*
AP ↔ FD	0.27	0.050
VJ ↔ FD	0.29	0.030

**Fig. 1:**
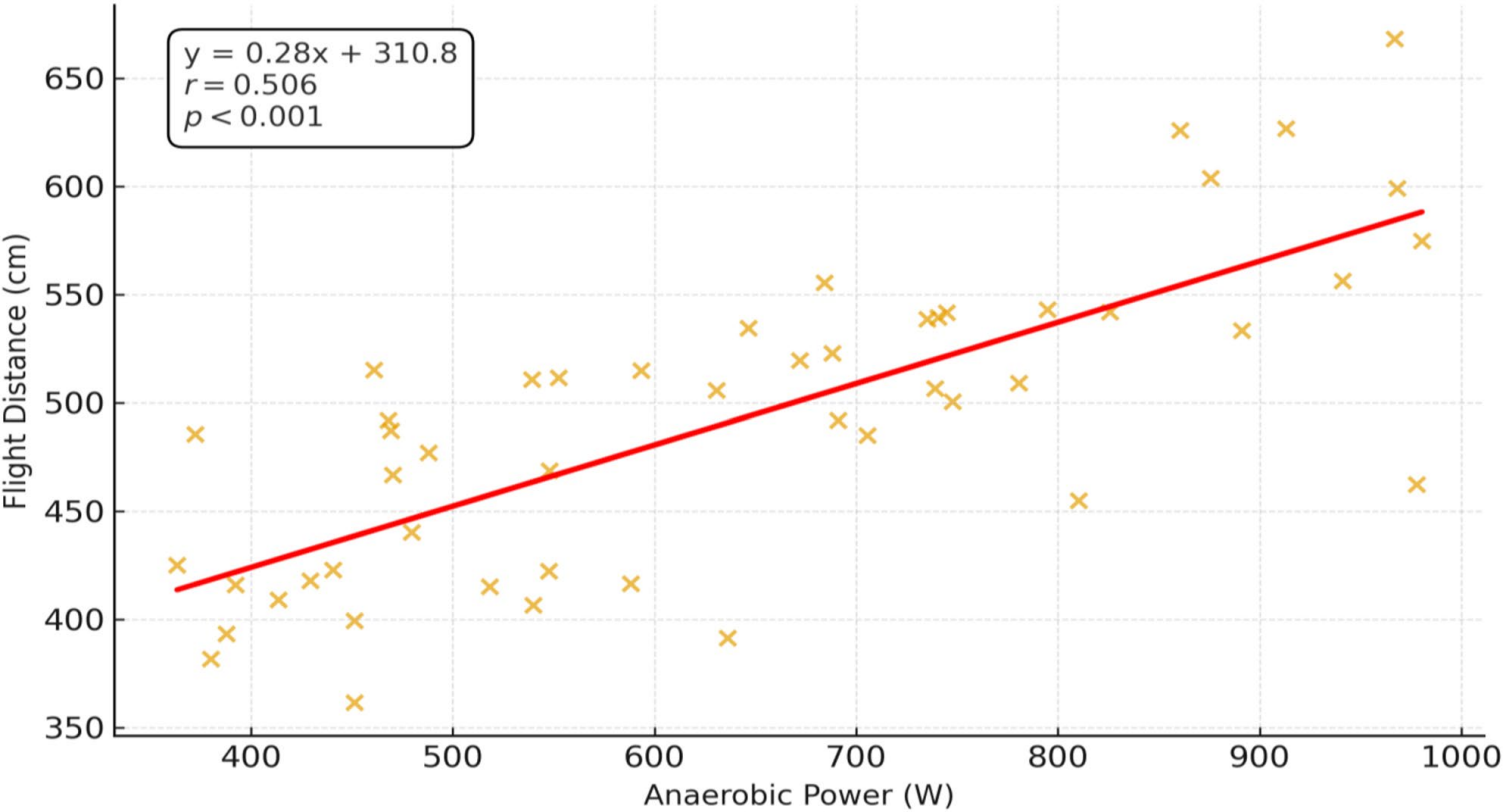
Bivariate correlation between AP and FD in adolescent swimmers

A significant positive association was observed between AP and FD, as illustrated in Fig. 1.

**Fig. 2:**
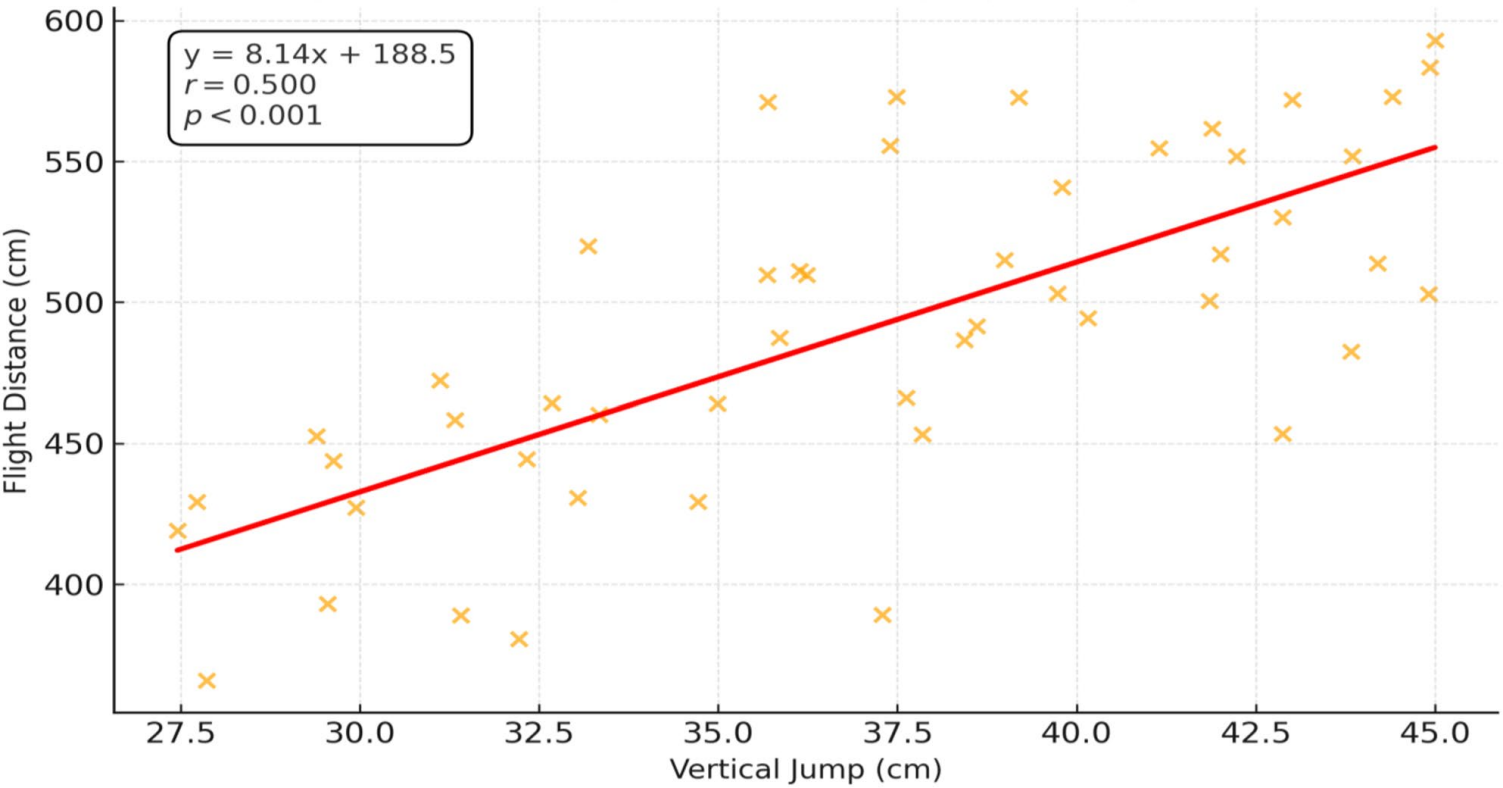
Bivariate correlation between VJ and FD in adolescent swimmers

Similarly, VJ performance showed a significant positive relationship with FD, as shown in Fig. 2.

A multiple linear regression analysis was conducted to examine the extent to which AP, age, and gender predicted FD. The overall regression model was statistically significant (F(3,50) = 22.63, *p*<0.001) and explained 55% of the variance in FD (R²=0.55). Age emerged as the strongest positive predictor of FD (β = 0.65, *p*<0.001), followed by gender (β = 0.32, *p* = 0.003), where male swimmers demonstrated greater distances. Interestingly, AP did not contribute significantly to the model once age and gender were controlled (β = 0.00, *p*=0.998). This lack of significance for AP suggests that its influence on flight distance is largely mediated by age-related physical maturation in adolescent swimmers. Furthermore, no multicollinearity was detected among the predictors, as all VIF values remained below 2, ensuring the stability of the regression coefficients (Table 5).

**Table 5:** Multiple regression results predicting flight distance

Predictor	B	SE	β	t	*p*	VIF
Constant	-126.17	60.93	—	-2.07	0.044	—
AP	0.00009	0.043	0.00	0.00	0.998	1.82
Age	29.30	5.15	0.65	5.69	0.001	1.53
Gender	37.99	12.27	0.32	3.10	0.003	1.24
Model fit: Adjusted R² = 0.55, F(3.50) ≈ 22.63, *p* < 0.001 Durbin–Watson: 1.14						

*FD* Flight Distance, *AP* Anaerobic power. Gender was coded as 1 = female and 2 = male. All predictors were entered simultaneously. No multicollinearity was detected (all VIF values < 2)

## Discussion

This study investigated the relationships between anaerobic power (AP), vertical jump (VJ), and flight distance (FD) during the start phase in adolescent swimmers. Male swimmers demonstrated higher AP, FD, and VJ values than female swimmers, with moderate-to-large effect sizes, indicating practically meaningful differences. Consistent with previous biomechanical studies [4, 16–22], both AP and VJ were positively correlated with FD, emphasizing the role of lower-limb power in swimming start performance.

Swimming start performance is a complex skill influenced by the integration of strength, power, and technique. The start consists of multiple phases—block, flight, underwater, and swimming [18, 23], each contributing to total performance time. Although this study focused solely on the flight phase, the observed relationships with AP and VJ indicate that the ability to generate high levels of force on land strongly contributes to effective take-off mechanics and increased horizontal velocity [19, 20]. Similarly, the relationship between VJ and FD supports the role of vertical jump as a practical index of explosive start performance [5, 24].

Initially, our bivariate analysis showed a positive correlation between AP, VJ, and FD. However, when integrated into a comprehensive multiple regression model alongside age and sex, AP lost its independent predictive power. This suggests that in adolescent swimmers, the variance previously attributed to raw power is better explained by chronological age and sex-related maturation. In this developmental stage, AP likely serves as a proxy for biological growth and cumulative training experience rather than an independent performance determinant. Furthermore, the lack of significance for land-based power in the final model may stem from the complex force transfer required on the starting block, which is not fully captured by vertical power tests. Therefore, our findings emphasize that for young athletes, technical mastery and maturational development are more influential for start performance than isolated land-based power. It should also be noted that anaerobic power was estimated using the Lewis formula, which is inherently dependent on body mass. This methodological characteristic likely contributed to the strong associations observed between anaerobic power and anthropometric variables and may partly explain the loss of independent significance in the regression model. Consequently, anaerobic power values derived from this formula may reflect combined effects of body mass and jump performance rather than isolated explosive capacity. Future studies may benefit from incorporating force–time or impulse-based jump metrics to provide a more independent representation of explosive strength in adolescent swimmers.

Our findings are partially consistent with Born et al. [6] and Akıl [25], who reported that swimmers with higher jump ability attained longer flight distances. Recent studies [7, 9] further highlight that land-based power assessments are valuable indicators of aquatic start potential. However, some studies [26, 27] have reported that improvements in vertical jump performance do not necessarily lead to significant changes in start performance, possibly due to insufficient integration of strength gains with technical training. Similarly, Benjanuvatra et al. [26] found that superior on-land jump ability did not always translate into longer start distances, particularly among less experienced swimmers, suggesting that block technique, take-off alignment, and underwater transitions may moderate the power performance relationship. This suggests that strength and technical drills should be combined to maximize performance benefits.

While lower-limb power is heavily emphasized in the literature as a key component of swimming start performance [4, 28], our regression findings deviate from this general expectation. In our model, AP did not significantly predict FD after controlling for variables such as age and sex. Conversely, age and sex emerged as significant predictors of FD. Specifically, the high beta coefficient for age highlights the decisive role of cumulative experience and long-term technical refinement in start performance. The lack of independent predictive power for AP deviates from the findings of studies such as [29], which may be attributed to the possibility that land-based AP assessments do not fully capture the complex force transfer mechanisms required during the actual aquatic phase. Furthermore, the substantial variance shared with age and sex may have statistically masked the independent contribution of AP within this specific adolescent cohort. These findings suggest that strategies to enhance start performance should prioritize individualized technical development and age-appropriate training interventions over generalized power-based conditioning alone.

The findings underscore the importance of integrating explosive power training with technical start practice. Coaches can utilize vertical jump and anaerobic power assessments to monitor readiness and tailor training interventions. Plyometric and resistance exercises, when combined with block-specific drills, may enhance power transfer and start efficiency. For adolescent swimmers, this integrative approach could support long-term performance development and improve competitive outcomes, where even minor improvements in start efficiency can translate into meaningful advantages in competition.

This study has certain limitations that must be considered. First, only the flight phase was analyzed, excluding block kinetics and underwater mechanics, which are critical to overall start efficiency. Second, the cross-sectional design limits our ability to draw causal inferences. Third, biological maturation status (e.g., Tanner stages or PHV) was not clinically assessed, which is a significant factor in performance variability during adolescence. Chronological age was therefore used as a proxy for developmental status; however, this approach may not fully capture individual differences in biological maturation during adolescence. Variability in maturation timing may partially mediate the observed relationships between anaerobic power, vertical jump performance, and flight distance, potentially influencing both strength expression and technical execution. Future studies should incorporate indirect maturation indicators, such as maturity offset or years from peak height velocity, to better disentangle the effects of biological development from training-related adaptations. Furthermore, despite standardized protocols, potential measurement errors in kinematic analysis could arise from slight variations in camera parallax or filming angles. Future research should utilize longitudinal designs and incorporate 3D biomechanical assessments alongside maturation status to better understand the transfer of land-based adaptations to aquatic performance.

## Conclusions

The findings of this study indicate that while anaerobic power and vertical jump height are positively associated with flight distance, age and sex are the strongest independent predictors of start performance in adolescent swimmers. These results emphasize that start efficiency should be viewed as a developmental process influenced by biological growth and technical refinement. For coaches and practitioners, these findings suggest that while vertical jump and anaerobic power assessments are valuable for monitoring physical readiness and talent identification, they should not be viewed in isolation. Training programs for young swimmers should prioritize the integration of targeted plyometric exercises with specific technical drills to optimize the transfer of land-based power into efficient start mechanics as the athletes mature.

## Data Availability

The data will be made available upon request.

## Abbreviations

AP: Anaerobic power
FD: Flight distance
VJ: Vertical jump
